# Identity Processing Style and Meaning in Life among Emerging Adults: Mediational Role of Commitment

**DOI:** 10.3390/ijerph19116585

**Published:** 2022-05-28

**Authors:** Natalia Czyżowska

**Affiliations:** Institute of Psychology, Pedagogical University of Kraków, 30-084 Krakow, Poland; natalia.czyzowska@up.krakow.pl

**Keywords:** identity style, commitment, meaning in life, emerging adulthood

## Abstract

Building one’s own identity and finding meaning in life are important tasks for emerging adults. Although many agree that both are essential in terms of the well-being and proper functioning of an individual, there is little empirical data concerning the relationship between the way young people process information about themselves and the feeling that their life has a purpose and value. Therefore, the aim of this study was to explore how identity processing styles (normative, diffuse-avoidant and informational) and two dimensions of meaning of life (presence and the search for meaning) are related. It was also assumed that identity commitment could play a mediating role between those variables. 183 emerging adults who participated in the study completed Identity Style Inventory (ISI-5) and Meaning in Life Questionnaire. The cross-sectional study design was used. Using Structural Equation Modeling revealed that commitment mediated the relationship between two of three identity styles (diffuse-avoidant and informational) and presence, but not search for or meaning in life. Normative identity style also negatively predicted the search for meaning in life. It seems that commitment might play an important role in meaning-making process, and as such, should be strengthened and encouraged when working with individuals at this stage in life.

## 1. Introduction

Emerging adulthood, between the ages of 18 and 29, is a period of intense changes, discernment and testing various possibilities [1]. Among the important challenges and developmental tasks of this period, there are defining who one is, and thus developing sense of identity and finding meaning in life [2,3]. In the classic approach proposed by Erikson [4], adolescence was the period in which the question of one’s own identity was primarily considered, but nowadays, due to social and economic changes taking place, it is noted that it is in the period of the emerging adulthood that young people have the most opportunities to explore their identity in different areas of life [5,6]. Although Erikson’s proposals were primarily theoretical and clinical, not empirical, they inspire many contemporary approaches in the study of identity issues [2,7].

One of such approaches is the model of identity formation proposed by Berzonsky [8,9], focusing on individual differences in processing and coding information and experiences important for the self, and thus in making personal decisions and solving problems. In this social-cognitive model, identity is viewed both as a construct and as a process. Berzonsky [10] claims that identity understood as a process allows the individual to direct and manage their resources in daily life. Identity as a construct is understood as a self-theory, a theory constructed by an individual about themself that serves as a referring point for interpreting self-relevant experiences and helping to make sense of them. Differences in processing self-relevant information and resolving identity conflicts are described as identity styles. There are three identity processing styles: normative, diffuse-avoidant and informational, which were described under the Berzonsky’s identity styles model [8,9]. Identity style preferences appear in late adolescence, though this does not mean a complete elimination of alternative strategies that may be used less frequently [11,12].

People who prefer a normative style tend to process identity issues fairly automatically, internalizing beliefs and values of significant others and adhering to established norms [8]. As research shows, a normative identity processing style is associated with conscientiousness and self-discipline, as well as the need for cognitive closure and structure, as well as limited ambiguity tolerance [13,14]. The diffusive-avoidant style is associated with delaying decision making and avoiding identity conflicts whenever possible. People for whom this style is dominant are driven primarily by hedonistic needs and short-term consequences. The diffusive-avoidant processing style is also positively related to impulsiveness, personal rumination and depression [15,16]. For people with a dominant informational style, it is characteristic to solve identity issues through active processing and evaluation of self-relevant information. This information style is associated with a tendency to explore identity options and broaden the knowledge about oneself. Research has shown that it is also associated with self-awareness, more adaptive coping strategies and wisdom [17,18,19]. Social-cognitive approach also pays attention to commitment, which can play an important role in the functioning of an individual by defining goals and strength of personal norms and beliefs [8,14]. Commitment gives a sense of purpose and direction to one’s actions, which can provide a frame of reference for monitoring, evaluating and regulating one’s own behavior [20]. It has been shown that there is a relationship between identity commitment and identity processing styles. Identity commitment correlates positively with the informative and normative style, and negatively with the diffuse-avoidant style [8,21].

In addition to developing a sense of identity, another important task faced by emerging adults is finding meaning in life. Development of sense of meaning in life is considered as a parallel process to development of sense of identity [22]. Contemporary researchers agree that the sense of meaning in life consists of three main components: significance; purpose; and comprehension. Significance, sometimes also called existential mattering, is a belief that one’s life matters. The purpose component relates to the feeling that undertaken actions are directed at worthwhile goals, and in accordance with one’s aspirations. Comprehension is a perception that one is able to understand what is happening to them in life, and integrate these experiences into a coherent whole [23,24]. These three components are reflected in the definition proposed by Steger [25], who describes meaning in life as a web of connections and interpretations that help people understand their experiences, plan for the future and move in the desired direction, and give them feeling that their lives count. Researchers also point out that the sense of meaning in life can be described on two dimensions: presence of meaning in life (how much an individual perceives their life as meaningful) and search for meaning, which reflects how much an individual seeks meaning [22]. The presence of meaning in life is positively associated with happiness, as well as with lower levels of anxiety and depression, while search for meaning is perceived as less beneficial to the functioning of an individual [22,26,27]. It is worth noting, however, that recently there have been studies that indicate that both the presence of meaning in life and searching for it can be good protective factors against suicide [28].

As the research results show, true self-knowledge and being in touch with one’s real self play an important role in the meaning-making process [29]. It seems unlikely that a person can be in touch with their real self without answering the question of who they are. Since identity in the aforementioned socio-cognitive approach by Berzonsky is understood as a self-theory, it seems that identity processing styles may also be important for the sense of meaning in life. However, so far this issue has not been studied in detail, and there are only single studies showing that there was a positive relationship between the presence of meaning in life and an informational identity style and a negative relationship between presence of meaning in life and a diffuse-avoidant style. A positive relationship was also shown between search for meaning in life and both normative and diffuse-avoidant style [18].

It is known that both the identity processing style and meaning in life are related to psychological well-being [30,31]. Ryff’s multidimensional model of psychological well-being refers to the eudaimonic tradition, which focuses on the development of the individual and living a satisfying, full life, and assumes that the sense of psychological well-being consists of six dimensions: (1) self-acceptance; (2) positive relations with others; (3) autonomy; (4) environmental mastery; (5) purpose in life; and (6) personal growth [32]. Regarding the relationship between meaning in life and psychological well-being, a positive relationship between presence of meaning and life and psychological well-being has been observed. A similar relationship was not noted in the case of search for meaning in life [31,33]. Researchers also noted that the relationship between identity styles and psychological well-being was mediated by identity commitment. Among the tested models, the best fit was the partial mediation model, in which both direct effects from identity styles to the well-being dimensions and indirect (mediated) effects from identity styles to psychological well-being through commitment were taken into account [30]. Furthermore, Berzonsky [8] pointed out that commitment can play an important role between identity processing style and outcome variables.

**Figure 1:**
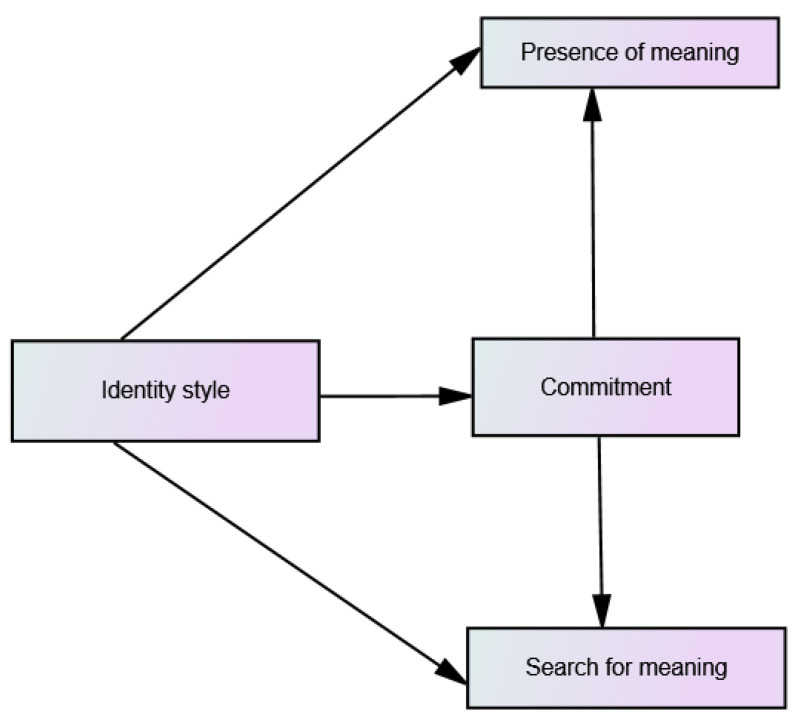
Hypothetical path model of the predictive relationships between identity style, commitment, presence of meaning and search for meaning in life.

The present study aimed to examine the relationship between the sense of meaning in life (both in presence and searching dimensions), and each of the three identity processing styles among emerging adults. It was assumed that, as in the case of psychological well-being, identity commitment will be a significant factor, influencing the relationships between other variables. In line with the main objective of the current study, a hypothetical path model assuming the above-mentioned relationships between the variables was created (see Figure 1).

## 2. Materials and Methods

### 2.1. Study Design, Participants and Recruitment

The study was planned as a cross-sectional study. The STROBE checklist for cross-sectional study was used [34] (entire checklist is available for review in Supplementary Materials). The research was conducted between January and September 2019. One hundred and eighty-three emerging adults (127 women and 56 men) who were recruited from three universities in Kraków (Lesser Poland Voivodeship, Poland) participated in the study (M_age_ = 20.81, SD = 1.84). All research participants were students or university graduates. Most of the participants (72%) lived in a large or medium city, while the remaining 28% lived in small towns or villages. None were married, but almost half declared having a partner (43%). None of the respondents had children. Recruitment took place via e-mail and through advertisements posted at universities and student forums. The study included people who were not treated psychiatrically, were not undergoing therapy and did not experience traumatic events in their lives in the last year, as it could significantly affect their level of sense of meaning in life. Participation in the study was voluntary, and the participants were informed in advance that they could resign from it at any time. Participants gave their written consent to participate after receiving oral and written information about the study. The study was conducted in accordance with the principles of the Declaration of Helsinki. The project was approved and financed from the funds earmarked for young scientists and doctoral students at the Faculty of Philosophy of the Jagiellonian University in Krakow.

### 2.2. Measures

The study used Polish language versions of the scales: Identity Style Inventory (ISI-5) [35], Meaning in Life Questionnaire [22] and a short questionnaire on demographic variables prepared by the researcher.

Identity Style Inventory (ISI-5) consists of 48 items (36 of which are diagnostic) that make up three subscales regarding identity styles: informational, normative and diffuse-avoidant, and a fourth scale that measures commitment (9 items in each). On a 5-point Likert scale, the respondents assess to what extent a given statement describes them (from “not at all like me” to “very much like me”). The Polish version of this scale was adapted by Senejko and Łoś [12]. The Cronbach alpha coefficients for the present study ranged from 0.69 to 0.81, so it can be considered as satisfactory.

Meaning in Life Questionnaire (MLQ) consists of 10 items that measure two dimensions of meaning in life: presence and search. Responses are provided on the 7-point Likert scale (from “absolutely untrue” to “absolute truth”). The Polish version of this scale was adapted by Kossakowska, Kwiatek and Stefaniak [36]. Cronbach alpha coefficients for the present study were 0.80 for the subscale measuring the presence of meaning in life, and 0.72 for search for meaning subscale.

### 2.3. Statistical Analysis

Listwise deletion was made to deal with missing data. In the first step, correlations between all variables were computed. In the next step, the hypothetical path models were tested using a structural equation model (SEM) in IBM SPSS AMOS 27. Hypothetical paths models were partial-mediation models in which each of three identity styles (normative, diffuse-avoidant and informational) predicted both commitment and meaning in life dimensions (presence and search). Meaning in life dimensions were also mediated by commitment. The maximum likelihood estimation method was used to test the adequacy of the proposed models. The adequacy of the model was evaluated using the following indices: non-significant *p*-value in χ^2^ test; the comparative fit index (CFI) above 0.95; the root mean square error of approximation (RMSEA); and the standardized root mean squared residual (SRMR) smaller than 0.08. To evaluate indirect relationships from identity styles to meaning in life dimensions through identity commitment, we used bias-corrected accelerated 95% confidence intervals (CI) calculated using 5000 bootstrapping re-samples [37]. When the CI for the unstandardized regression coefficient does not include zero, an indirect mediated effect is considered to be significant [38]. It is indicated that the minimum sample size to perform SEM is *n* = 100–150 [39]. Referring to Kline’s [40] recommendation that the *n*:q ratio should be 20 to 1, the sample size (*n* = 183) was sufficient to perform a specified path model analysis.

## 3. Results

Descriptive data and Pearson’s correlation matrix among the identity styles, commitment and both dimension of meaning in life (presence and search) are presented in Table 1. Presence of meaning in life was positively associated with informational identity style and commitment, and negatively associated with both a diffuse-avoidant and normative style. Search for meaning was positively correlated with informational style, and negatively correlated with normative style.

**Figure 2:**
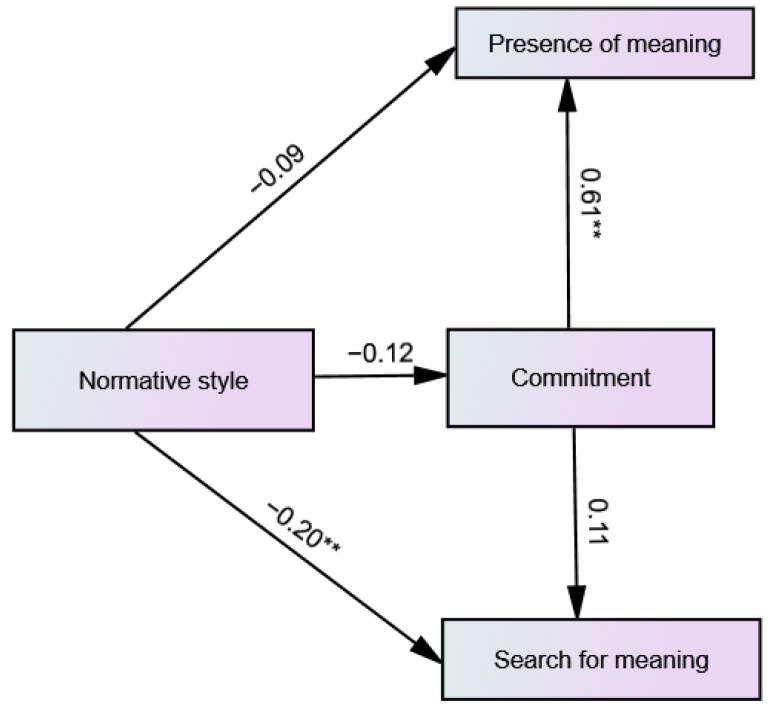
Path diagram of the predictive relationships between normative identity style, commitment, presence of meaning and search for meaning, including the standardized path coefficients. ** *p* < 0.01.

The hypothesized model of the predictive relationships between normative identity style, commitment, presence of meaning and search for meaning in life was found to be a good fit for the data [χ^2^_(1)_ = 1.93, *p* = 0.164, CFI = 0.99, RMSEA = 0.071 (90% CI 0.00–0.22), SRMR = 0.024]. As shown in Figure 2, only paths from commitment to presence of meaning in life and from normative identity style to search for meaning in life were significant. Commitment positively predicted presence of meaning in life and normative identity style negatively predicted search for meaning in life.

**Figure 3:**
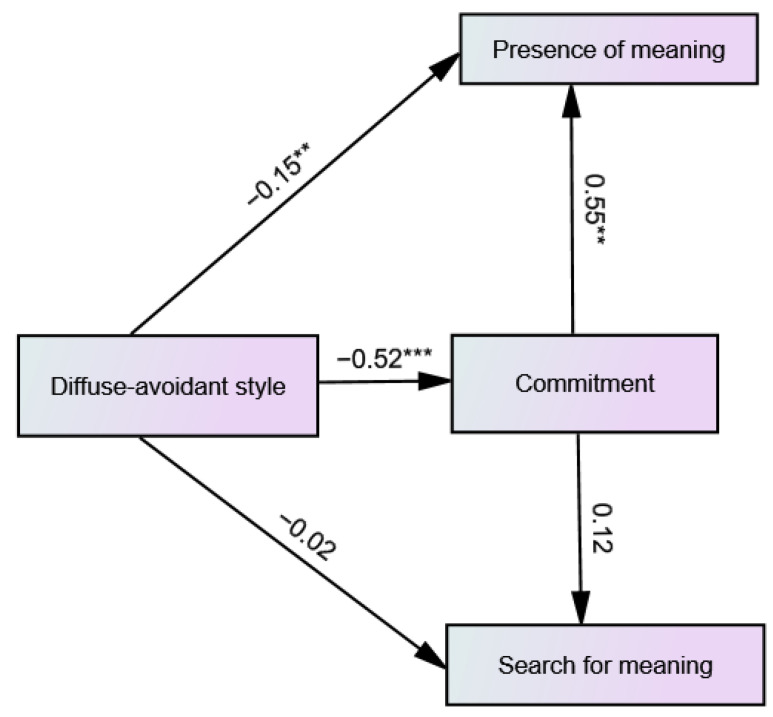
Path diagram of the predictive relationships between diffuse-avoidant identity style, commitment, presence of meaning and search for meaning including the standardized path coefficients. ** *p* < 0.01. *** *p* < 0.001.

The hypothesized model of the predictive relationships between diffuse-avoidant identity style, commitment, presence of meaning and search for meaning in life was also found to be a good fit for the data [χ^2^_(1)_ 1.26, *p* = 0.262, CFI = 0.99, RMSEA = 0.037 (90% CI 0.00–0.02), SRMR = 0.019]. As shown in Figure 3, paths from diffuse-avoidant style to presence of meaning in life and commitment and from commitment to presence of meaning in life were significant. The diffuse-avoidant style negatively predicted the presence of meaning in life and commitment. Commitment positively predicted presence of meaning in life. For the commitment mediated path from the diffuse-avoidant style to presence of meaning in life, the indirect mediated effect was significant (β = −0.28, SE = 0.046, 95% CI −0.372 to −0.192).

**Figure 4:**
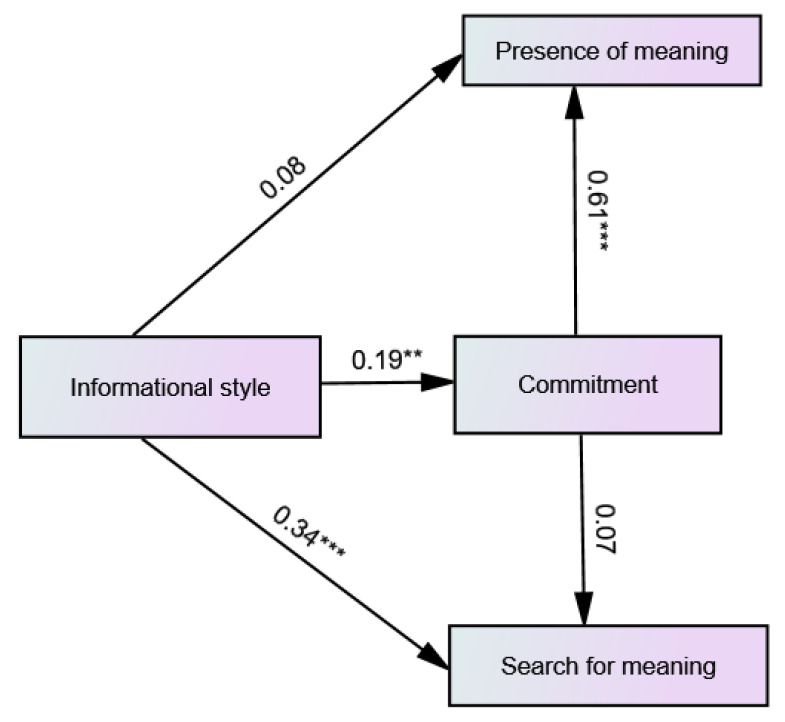
Path diagram of the predictive relationships between informational identity style, commitment, presence of meaning and search for meaning, including the standardized path coefficients. ** *p* < 0.01. *** *p* < 0.001.

The hypothesized model of the predictive relationships between informational identity style, commitment, presence of meaning and search for meaning in life did not fit as well with the data as the two previous models [χ^2^_(1)_= 2.68, *p* = 0.101, CFI = 0.98, RMSEA = 0.09 (90% CI 0.00–0.24), SRMR = 0.027]. As shown in Figure 4, paths from informational style to search for meaning in life and commitment and from commitment to presence of meaning in life were significant. Informational style positively predicted search for meaning in life and commitment and commitment positively predicted presence of meaning in life. In the commitment mediated path from the informational style to presence of meaning in life, the indirect mediated effect was significant (β = 0.14, SE = 0.055, 95% CI 0.032 to 0.254).

## 4. Discussion

The current research shows that first, there is a negative relationship between the normative identity style and both dimensions of meaning in life: presence and search. However, as shown by structural equation modelling, normative identity style negatively predicts only search of meaning in life. It is inconsistent with previous research that indicates a *positive* relationship between normative identity style and both the presence of meaning and search of meaning in life [18]. Nevertheless, the negative relationship between normative processing identity style and search for meaning observed here seems to be consistent with the identity style theory. According to the theory, people with a dominant normative identity style almost automatically adopt certain norms and beliefs from significant others [8,9], and thus might not be eager to actively explore on their own not only values but also meaning in life. Second, a negative relationship between the diffuse-avoidant identity style and presence of meaning in life was found; this result is consistent with both previous research and the identity processing style theory [8,9]. The data obtained so far show that the diffuse-avoidant style is negatively related to all six dimensions of psychological well-being [30], including purpose in life, which is also one of the elements of meaning in life [23,24]. People with a predominant diffuse-avoidant style are considered impulsive, following hedonistic needs, which do not seem to support the meaning-making process. In the present research, the diffuse-avoidant style negatively predicts both the presence of meaning in life and commitment, a result also reported by other researchers [21]. No relationship was found between diffuse-avoidant style and the second dimension of meaning in life, search for meaning, although previous studies indicated a positive relationship between those variables [18]. The latter result seems perplexing given the characteristics of people with diffuse-avoidant style, derived from the identity processing style theory, who have limited self-awareness, delay making decisions as long as possible, and when they do have to make them, are guided mainly by situational demands [41]. Thus, the results obtained in the current study seem to better fit the theory. Finally, informational identity style positively correlates with both dimensions of the sense of meaning in life, presence and search, though as shown by structural equation modeling, predicts only search for meaning. People who prefer informational identity style are motivated to improve their knowledge about themselves and are characterized by open-mindedness [15]. This attitude seems to favor exploration in various areas of life, including the search for the sense of it.

Moreover, the obtained results showed that there is a positive relationship between commitment and presence of meaning in life. Commitment also has proven to be a good predictor of this dimension of meaning in life. This result seems to be particularly interesting, as in previous studies concerning the relationships between identity styles and sense of meaning in life, commitment was only used in correlation analyses, and was not included in the tested models [18]. Here, it was assumed that, as in the case of psychological well-being, commitment would mediate between identity styles and meaning in life. The mediational role of commitment was confirmed only for two out of three identity styles, diffuse-avoidant and informational, and for only one of two dimensions of meaning in life, presence of meaning, but not the search for it. However, the types of mediation for those two identity styles are different. Referring to the “causal steps” approach to mediation proposed by Baron and Kenny [42], when the strength of the relationship between the independent variable (i.e., identity style) and dependent variable (i.e., presence of meaning in life) is weakened after introducing into the equation the mediational variable (i.e., commitment), we are dealing with a partial mediation. This is the case of the relationship between diffuse-avoidant style and presence of meaning in life. Alternatively, when introducing a mediator into the equation reduces the structural relationship between the independent (i.e., identity style) and dependent variable (i.e., presence of meaning in life) to non-significant then it is a complete mediation. Such a relationship has been observed between informational style and presence of meaning in life. This pattern of results could suggest that in the case of diffuse-avoidant style commitment might be “cancelling out” the negative influence of such a processing style on the presence of meaning in life, while in the case of the informational style commitment, as one of its features, might be the sole vehicle for achieving meaning in life.

The obtained results suggest that it is commitment that may be of great importance for establishing meaning in life. Researchers indeed note that it is vital for meaning-making in a life-time perspective, although they focused primarily on the elderly [43]. Such observation might be valuable for counsellors, as encouraging one’s personal engagement and pursuing individual goals deemed as worthwhile and important could strengthen the perceived meaning of life. It is worth noting that obligations anchor a person in a specific set of roles (such as partner, parent or employee), and although emerging adults often have not yet undertaken them formally or legally, the very fact of making life commitments (e.g., in the area of romantic relationships or professional goals) increases their sense of coherence [2,44]. Thus, commitment might help to integrate one’s own experiences into the coherent whole, which is one of the components of meaning in life. This study seems to confirm what has already been pointed out, that promoting a coherent sense of identity along with stable and strong commitments will have a positive impact on the functioning of young adults [44].

### Limitations and Future Directions

So far, there have been only few studies that focus on the relationship between identity processing style and meaning in life. As there are some discrepancies between the results reported here and obtained in the previous studies, more research is needed to define more precisely and further explore the relationships between the above-mentioned constructs. Both the current and previous studies were conducted on a relatively small sample of emerging adults (less than two hundred people), so it would be advisable to repeat it with a larger group of participants. Another limitation of the current study is the uneven number of women and men, which made conducting multi-group analyses impossible. It cannot be ruled out that that gender might be a factor affecting the studied relationships, and therefore it should be considered in future studies. The group taking part in the study was also quite homogeneous, as it consisted of students and people who graduated from university, so it would be worthwhile to check whether the obtained pattern of relationships will be observed in a group of emerging adults who did not choose to pursue higher education. It should also be noted that the research was conducted only on the Polish sample, which means that caution should be exercised in order to generalize the results for the entire population of emerging adults. This is because social and cultural conditions may influence the studied variables [26]. Due to the single and simultaneous measurement of all the studied variables, it was not possible to infer the developmental impact of identity processing styles. Future studies should therefore take into account the longitudinal measurement of the described variables. Considering that both identity styles and sense of meaning in life are related to psychological well-being, and there has been no research so far that would consider all these constructs together and describe the relationships between them, it seems that in future research it would be worth undertaking this topic. Due to the fact that the research was one of the first to focus on the mediating role of commitment between identity styles and meaning in life, further research on this issue is necessary.

## 5. Conclusions

Based on the data collected and analyzed, the following conclusions can be drawn:

1. There is a negative relationship between normative identity style and both dimensions of meaning in life: presence of meaning and search for meaning in the group of emerging adults. Normative identity style negatively predicts search for meaning in life, but not presence of meaning.
2. There is a negative relationship between diffuse-avoidant identity style and the presence of meaning in life in the group of emerging adults.
3. There is a positive relationship between informational identity style and both dimensions of meaning in life in the group of emerging adults. Informational style positively predicts search for meaning in life.
4. Normative identity style negatively predicts search for meaning in life, and informational style positively predicts this dimension of meaning in life in the group of emerging adults.
5. Commitment predicts presence of meaning in life. What’s more, commitment partially mediated relationship between diffuse-avoidant identity style and the presence of meaning in life and a fully mediated relationship between informational identity style and presence of meaning in life.
6. Commitment seems to play a significant role in meaning-making among emerging adults.

## Figures and Tables

**Table 1:** Means, standard deviations and correlations among all study variables.

Variable	M	SD	Range of Possible Scores	1.	2.	3.	4.	5.	6.
Normative style	20.68	4.47	5.00–45.00	-					
2. Diffuse-avoidant style	21.52	5.25	5.00–45.00	0.27 **	-				
3. Informational style	36.19	4.19	5.00–45.00	−0.32 **	−0.12	-			
4. Commitment	31.32	5.30	5.00–45.00	−0.12	−0.49 **	0.21 **	-		
5. Presence of meaning in life	21.51	5.11	5.00–35.00	−0.17 **	−0.40 **	0.22 **	0.61 **	-	
6. Search for meaning in life	25.92	3.89	5.00–35.00	−0.21 **	−0.09	0.33 **	0.15	0.03	-

Statistically significant results; ** *p* ≤ 0.01.

## Data Availability

The data that support the findings of this study are available from the corresponding author, upon reasonable request.

## Supplementary Materials

STROBE checklist for cross-sectional studies can be found at: https://www.strobe-statement.org/checklists/ (accessed on 19 May 2022)
